# Multiple Evolutionary Events Involved in Maintaining Homologs of *Resistance to Powdery Mildew 8* in *Brassica napus*

**DOI:** 10.3389/fpls.2016.01065

**Published:** 2016-07-21

**Authors:** Qin Li, Jing Li, Jin-Long Sun, Xian-Feng Ma, Ting-Ting Wang, Robert Berkey, Hui Yang, Ying-Ze Niu, Jing Fan, Yan Li, Shunyuan Xiao, Wen-Ming Wang

**Affiliations:** ^1^Rice Research Institute and Key Laboratory for Major Crop Diseases, Sichuan Agricultural University at WenjiangChengdu, China; ^2^ Institute for Bioscience and Biotechnology Research and Department of Plant Science and Landscape Architecture, University of Maryland, College Park, College ParkMD, USA; ^3^College of Agronomy, Sichuan Agricultural University at WenjiangChengdu, China

**Keywords:** *RPW8*, *Brassica rapa*, *Brassica oleracea*, paralog, ortholog, powdery mildew, polymorphism

## Abstract

The *Resistance to Powdery Mildew 8* (*RPW8*) locus confers broad-spectrum resistance to powdery mildew in *Arabidopsis thaliana*. There are four *Homologous to RPW8*s (*BrHR*s) in *Brassica rapa* and three in *Brassica oleracea* (*BoHR*s). *Brassica napus* (*Bn*) is derived from diploidization of a hybrid between *B. rapa* and *B. oleracea*, thus should have seven homologs of RPW8 (*BnHR*s). It is unclear whether these genes are still maintained or lost in *B. napus* after diploidization and how they might have been evolved. Here, we reported the identification and sequence polymorphisms of *BnHR*s from a set of *B. napus* accessions. Our data indicated that while the *BoHR* copy from *B. oleracea* is highly conserved, the *BrHR* copy from *B. rapa* is relatively variable in the *B. napus* genome owing to multiple evolutionary events, such as gene loss, point mutation, insertion, deletion, and intragenic recombination. Given the overall high sequence homology of *BnHR* genes, it is not surprising that both intragenic recombination between two orthologs and two paralogs were detected in *B. napus*, which may explain the loss of *BoHR* genes in some *B. napus* accessions. When ectopically expressed in *Arabidopsis*, a C-terminally truncated version of *BnHRa* and *BnHRb*, as well as the full length *BnHRd* fused with YFP at their C-termini could trigger cell death in the absence of pathogens and enhanced resistance to powdery mildew disease. Moreover, subcellular localization analysis showed that both BnHRa-YFP and BnHRb-YFP were mainly localized to the extra-haustorial membrane encasing the haustorium of powdery mildew. Taken together, our data suggest that the duplicated BnHR genes might have been subjected to differential selection and at least some may play a role in defense and could serve as resistance resource in engineering disease-resistant plants.

## Introduction

Plant resistance (*R*) genes have been widely exploited in crop breeding to raise disease resistant cultivars for minimizing crop losses to pathogens worldwide. Based on the putative protein structures, *R* genes encode three classes of proteins (Xiao et al., 2008). The first class of R proteins belong to the nucleotide-binding site and leucine-rich-repeat (NBS-LRR or NLR) superfamily that act as the intracellular immune receptors capable of recognizing specific pathogen effectors and subsequently triggering defense responses (Bonardi et al., 2012). Most functionally characterized plant R proteins fall into this class. Based on the presence of an N-terminal coiled-coil (CC) or the Toll/interleukin1 receptor (TIR) domain, NLR proteins are further classified into CNL and TNL (Meyers et al., 2003). The second class encodes proteins possess an extracellular LRR (eLRR) domain such as receptor-like kinases (RLKs) and surface receptor-like transmembrane proteins (RLPs; Jones et al., 1994; Dangl and Jones, 2001). The third class of *R* genes is designated atypical because they encode novel proteins or proteins with a novel domain that are distinct from NLR or eLRR type R proteins. For example, the tomato R protein Pto is a Serine/Threonine kinase mediating resistance to different strains of *Pseudomonas syringae* (Martin et al., 1993; Swiderski and Innes, 2001). The wheat R protein PM21 is also a Serine/Threonine kinase mediating broad-spectrum and durable resistance to powdery mildew disease in wheat (Cao et al., 2011). Another wheat R protein LR34 is a putative ATP-binding cassette transporter conferring resistance to multiple fungal pathogens (Krattinger et al., 2009). The rice R protein Xa27 contains two putative transmembrane domains and is specifically induced at the infection site initiating resistance to *Xanthomonas oryzae* pv. *oryzae (Xoo*) strains harboring AvrXa27 (Gu et al., 2005, 2009).

The *Arabidopsis* R proteins RPW8.1 and RPW8.2 are also considered to be atypical because of their putative unique protein structure, both are small (18–20 kDa) with a putative N-terminal transmembrane (TM) domain or signal peptide and one or two coiled-coils (CCs; Xiao et al., 2001). *RPW8.1* and *RPW8.2* (referred to as *RPW8* in later text unless otherwise indicated) are tandemly arrayed with three *Homologous to RPW8*s (*HR*s), *HR1, HR2*, and *HR3* at the *RPW8* locus from the *Arabidopsis* accession Ms-0. In the accession Col-0, however, *RPW8.1* and *RPW8.2* were replaced by *HR4* (Xiao et al., 2001). *RPW8* and its family members may have evolved from an *HR3*-like progenitor gene via gene duplication followed by diversification (Xiao et al., 2004). Intriguingly, RPW8 and its homologs show sequence homology to the N-terminal domain of a unique clade of NB-LRRs in many plant genomes, and these RPW8-domain-containing NB-LRRs are defined as RPW8-NB-LRR (RNL; Bonardi et al., 2011; Collier et al., 2011; Shao et al., 2014; Zhang et al., 2016). *RNL* genes are clustered as a sister clade to the clade of *CNL* genes in the phylogenetic tree of *NLR* genes from five Brassicaceae species (Zhang et al., 2016). Unlike the *CNL* or *TNL* genes that account for the vast majority of the *NLR* genes across angiosperm plants, *RNL* genes belong to a small group that contains less than 10 members (Shao et al., 2014; Zhang et al., 2016). Given this evolutionary link to RNLs, how RPW8 in *Arabidopsis* confers disease resistance to powdery mildew is of particular interest. Our recent work showed that *RPW8.2* expression is induced by powdery mildew infection and the RPW8.2 protein is specifically targeted to the extra-haustorial membrane (EHM) encasing the fungal feeding structure, the haustorium, to activate defense against powdery mildew (Wang et al., 2009). Accordingly, adequate expression and precise EHM-specific localization of RPW8.2 are required for cost-effective resistance to powdery mildew (Wang et al., 2009, 2010). RPW8.1 is functionally distinguished from RPW8.2 in triggering cell death and disease resistance. While RPW8.2 confers resistance to powdery mildew, ectopic expression of RPW8.1 leads to enhanced resistance to both powdery mildew and downy mildew (Ma et al., 2014).

*Brassica napus* is an important oilseed crop in the world, providing approximately 13% of the world’s supply of vegetable oil (Hajduch et al., 2006). Its allotetraploid genome (AACC, *2n* = 38) is thought to have originated from a spontaneous diploidization after hybridization between *Brassica rapa* (AA, *2n* = 20) and *Brassica oleracea* (CC, *2n* = 18; Parkin et al., 2005). Previously, two distinct loci containing *HR* genes were identified in *Brassica* species. One locus contains three tandemly arrayed *HR* genes from both *B*. *oleracea* and *B*. *rapa*, namely, *BoHRa* (AY225587), *BoHRb* (AY225588), *BoHRc* (AY225589), *BrHRa, BrHRb*, and *BrHRc* (AY225586). The other locus contains only one *HR* gene, *BrHRd* (AY225590) from *B. rapa* (Xiao et al., 2004). All these *Brassica* genes have the highest similarity to the *Arabidopsis HR3* gene (Xiao et al., 2004). However, whether these genes have all been maintained in the *B. napus* genome after hybridization and diploidization has not been determined.

In the present study, to understand the maintenance and possible functional divergence of *BnHR* genes in *B. napus*, we first identified and sequenced these genes, and analyzed the sequence polymorphisms among all the *BnHR* genes from *B. napus*. We then introduced them into the powdery mildew-susceptible *Arabidopsis* accession Col-gl (Col-0 harboring the glabrous mutation 1) and examined their protein subcellular localization and ability to activate disease resistance against powdery mildew. Our data indicate that multiple evolutionary events, including gene loss, point mutation, insertion, deletion and intragenic recombination, were involved in maintaining *BnHR* genes in the *B. napus* genome after hybridization and diploidization. Moreover, ectopic expression of some *BnHR* genes leads to cell death and enhanced resistance to powdery mildew, suggesting that they could be valuable for engineering disease-resistant rapeseed plants.

## Materials and Methods

### Plant Materials

Eighty-eight accessions of *B. napus* from our rapeseed-breeding resource were selected for sequence determination for different *BnHR* genes. Nucleotide sequences of *BrHRa, BrHRb, BrHRc, BrHRd, BoHRa, BoHRb*, and *BoHRc* from a previous study determined in Xiao et al. (2004) were used as control in sequence variation analysis. By comparison with the reported homologs of *RPW8* in *B*. *rapa* (*BrHR*) and *B. oleracea* (*BoHR*), we designated *BnHR*(*Br*) and *BnHR*(*Bo*) for homologs of *RPW8* in *B. napus* closed to *BrHR* and *BoHR*, respectively.

### Gene Amplification and Sequence Analysis

Degenerate primer BamBnHRR1 was paired with BamBnHRaF, BamBnHRbF, or BamBnHRcF to amplify *BnHRa*(*Br*)/*BnHRa*(*Bo*), *BnHRb*(*Br*)/*BnHRb*(*Bo*), or *BnHRc*(*Br*)/*BnHRc*(*Bo*), respectively (Supplementary Table S1). Gene-specific primers BamBrHRdF and BamBrHRdR were used to amplify *BnHRd*. PCR products were purified and sequenced from both strands. Sequences with variation from *BrHR*s or *BoHR*s have been submitted to GenBank and assigned accession numbers listed in the Supplementary Table S2. DNA sequences were aligned using AlignX function of Vector NTI Suite (Invitrogen) and corrected manually. Amino acid sequences were deduced from the nucleotide sequences by Vector NTI and aligned by AlignX. DnaSP version 5.10.1 was used for calculation of nucleotide polymorphism and divergence, as well as Tajima’s D test (Rozas, 2009). Synonymous (Ks) and non-synonymous substitution (Ka) rates between *BnHR* alleles and their *BrHR*/*BoHR* ancestors were calculated for an alignment of the coding sequences after gapped sites were removed. Calculations were made by the yn00 program of PAML version 4.9a under PAMLX graphical user interface (Yang, 2007; Xu and Yang, 2013).

### Transgenic and Microscopy Analyses

Genomic DNA isolated from the accessions R7, RS-2, and RS-3 was used as template to amplify BnHR genes. We chose these three accessions because they are elite germplasm exhibiting field-resistance against powdery mildew and downy mildew, and thus have been widely exploited in breeding programs. The primer BamHRR1 was paired with BrHRaF, BrHRbF, or BrHRcF to amplify *BnHRa*(*Br*), *BnHRb*(Br), or *BnHRc*(*Br*), respectively. The amplified fragments were cloned into the binary vector pP2Y3′ (Wang et al., 2013) *Bam*HI site resulting in constructs expressing the full length BnHR proteins fused with YFP at the C-terminus driven by the *RPW8.2* promoter. For making constructs expressing the C-terminally truncated proteins, primer pairs BrHRaF/BrHRaT1R, BrHRbF/BrHRbT1R, and BrHRcF/BrHRcT1R were used to amplify *BnHRat*(*Br*), *BnHRbt*(Br), and *BnHRct*(*Br*), respectively (Supplementary Table S1). The amplified fragments encoding the first 164 amino acid (aa) residues, the number of aa close to that of RPW8, were cloned into the binary vector pP2Y3′ *Bam*HI site resulting in constructs expressing the C-terminal truncated BnHR proteins fused with YFP driven by the *RPW8.2* promoter. Then, these constructs were introduced into Col-gl via Agro-mediated floral dipping (Clough and Bent, 1998). At least 15 independent T_1_ lines for each construct were generated and tested for their spontaneous cell death and disease phenotypes in response to *Golovinomyces cichoracearum* UCSC1. The *Arabidopsis* line S5 containing a single copy of *RPW8.1* and *RPW8.2* under control of their native promoters was used as resistant reference (Xiao et al., 2003). The statistical significance in the number of spores per mg fresh leaf was examined by *post hoc* comparisons [Tukey’s Honestly Significant Difference test by using DPS (Data Processing System) statistical software version 7.05]. For examination of EHM-localization, 10 plants from three representative T_2_ lines for each construct were inoculated with the tobacco powdery mildew stain *G*. *cichoracearum* SICAU1 that were maintained on tobacco plants (Zhang et al., 2015). Then, examination of EHM-localization was repeated on T_3_ lines. Laser Scanning Confocal Microscopy images were acquired following the user’s manual when using a Nikon A1 microscope. All pictures presented in the figures were projections from Z-stacks of 10–50 images, unless otherwise indicated. The image data were processed using NIS-Elements viewer and Adobe Photoshop.

## Results

### Isolation of Homologs of *RPW8* from *B. napus*

**FIGURE 1:**
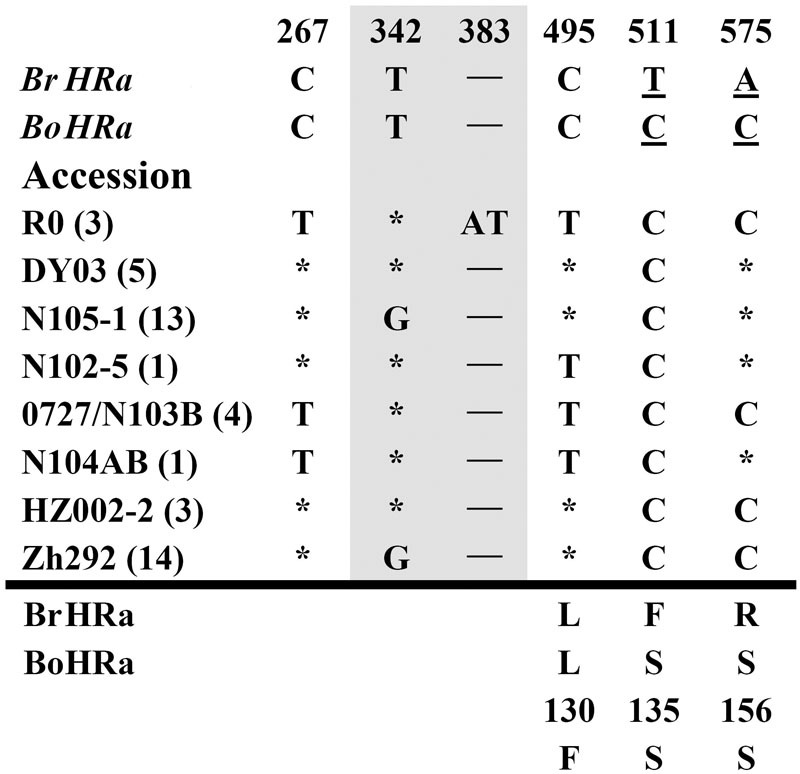
**Polymorphic sites of *BnHRa*(*Br*) aligned against the *BrHRa* allele.** An asterisk indicates an identical nucleotide to that of *BrHRa*. Shaded letters are the substitutions in the intron. The numbers at the top indicate the nucleotide position from the start codon of *BrHRa* allele. Amino acid replacements resulting from nucleotide substitutions are indicated at the bottom. *BoHRa* was included to compare the variable sites in *BnHRa*(*Br*). Underlined letters indicate the polymorphism sites between *BrHRa* and *BoHRa*.

**FIGURE 2:**
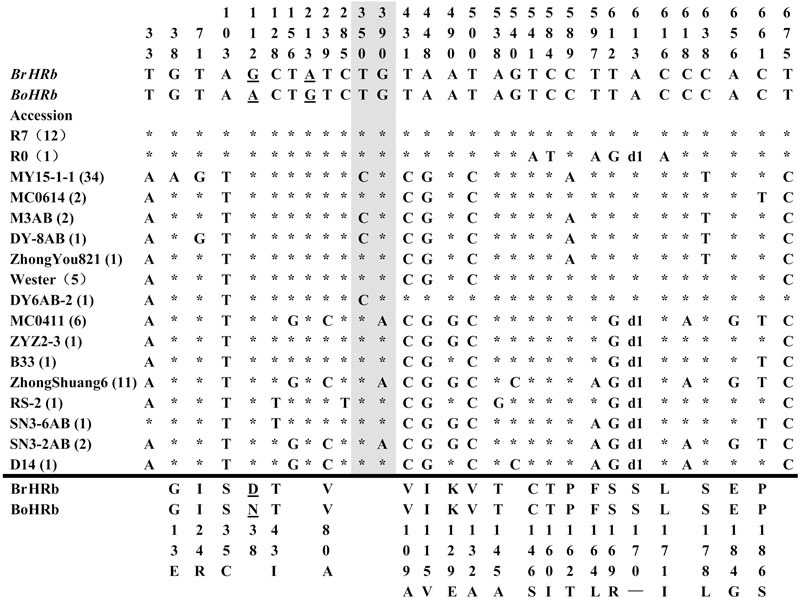
**Polymorphic sites of *BnHRb*(*Br*) aligned against the *BrHRb* alleles.** An asterisk indicates an identical nucleotide. Shaded letters are the substitutions in the intron. The numbers at the top indicate the nucleotide position from the start codon of *BrHRb* alleles. Amino acid replacements resulting from nucleotide substitutions are indicated at the bottom. d1, a deletion of AGT. The two sites telling the difference between *BrHRb* and *BoHRb* are underlined.

**FIGURE 3:**
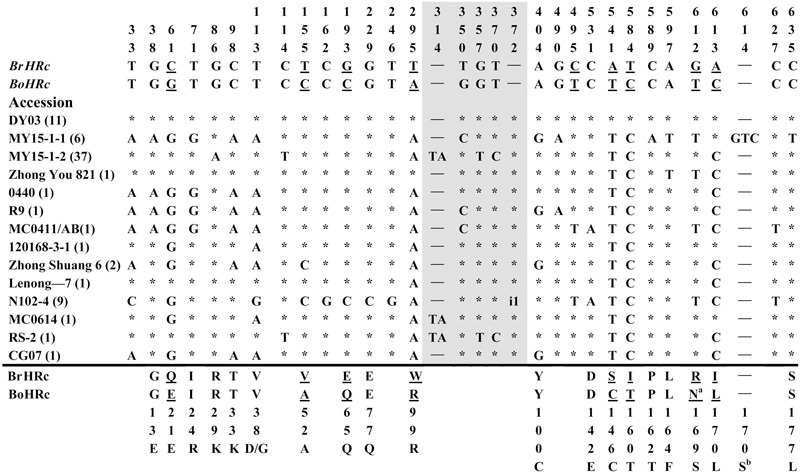
**Polymorphic sites of *BnHRc*(*Br*) aligned against the *BrHRc* allele.** An asterisk indicates an identical nucleotide and a dash indicates a gap. Shaded letters are the substitutions in the intron. The numbers at the top indicate the nucleotide position from the start codon of *BrHRc* allele. Amino acid replacements resulting from nucleotide substitutions are indicated at the bottom. Underlined letters indicate the polymorphism sites between *BrHRc* and *BoHRc*. Two insertions were detected, one is TA at nt 314 in the intron from 39 accessions; and the other is presented as i1, an insertion of 31 bp (TTTTTTTTCAATAAATGTTTTTATTTTGTTT) in the intron of HRc from nine accessions. ^a^The code for this residue in *BoHRc* is AAT (encoding N), but *AGG* (encoding R) in *BrHRc* and changed to AGT (encoding S) in some accessions. ^b^The insertion of GTC between nt 614 and 615 led to an inserted S (AGT, GT add to the upstream A forming AGT) at aa position 170 in this accession, while aa 171 changed from I to L (CTC, C add to the downstream TC forming CTC) as those at aa 170.

**FIGURE 4:**
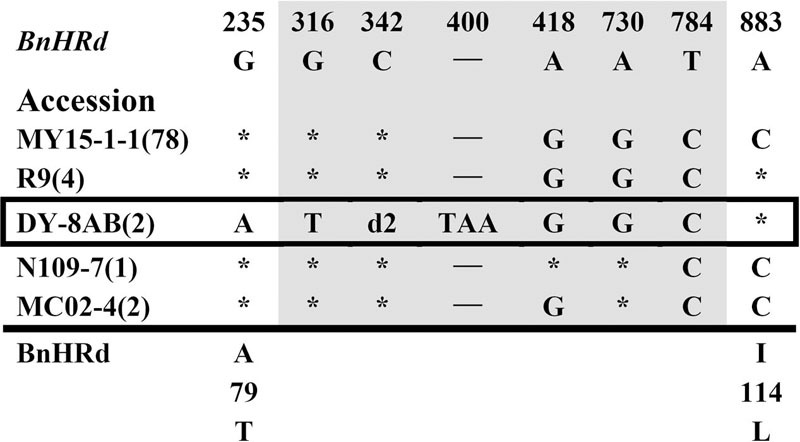
**Polymorphic sites of *BnHRd* aligned against the *BrHRd* allele.** An asterisk indicates an identical nucleotide and a dash indicates a gap. Shaded letters are the substitutions in the intron. The numbers at the top indicate the nucleotide position from the start codon of *BrHRd* allele. Amino acid replacements resulting from nucleotide substitutions are indicated at the bottom. d2 represents a deletion of 10 bp (CATGAAAAAG).

To isolate homologs of *RPW8* from *B. napus*, we exploited the evolutionary relationship between *B. napus* and its two ancestors *B*. *rapa* and *B*. *oleracea*. By using primers designed from a previous report (Xiao et al., 2004), we conducted PCR amplification of *BnHR* genes from 88 accessions of *B. napus*. As shown in Supplementary Table S3, we successfully amplified *BnHRa*(*Bo*) from 77 accessions with no sequence variation from that of *BoHRa*, whereas, we amplified *BnHRa*(*Br*) from 44 accessions with sequence variation from that of *BrHRa* (**Figure 1**), suggesting that *BrHRa* was lost in half of the accessions tested and the maintained ones are relatively variable. As for *BnHRb*, we were successful in amplification from 83 accessions with 12 sequences identical to *BrHRb*, but none was identical to *BoHRb* (Supplementary Table S3; **Figure 2**). Because two single nucleotide polymorphisms (SNPs) distinguish *BrHRb* and *BoHRb*, these data indicate that all amplified *BnHRb* alleles are derived from *BrHRb* of *B*. *rapa*. We also successfully amplified *BnHRc*(*Bo*) from 63 accessions, all were identical to that of *BoHRc*, whereas, we got *BnHRc*(*Br*) from 74 accessions of which 11 were identical to *BrHRc* (Supplementary Table S3; **Figure 3**). *HRd* is derived from *B. rapa* and is also most similar to *HR3*, a homolog of *RPW8* in *Arabidopsis* that is the hypothetical progenitor for all members of *RPW8* family (Xiao et al., 2004). We successfully amplified *BnHRd* from all 88 *B. napus* accessions, of which one was identical to *BrHRd* and 87 had sequence variation from that of *BrHRd* (Supplementary Table S3; **Figure 4**). Collectively, we successfully amplified at least three *BnHR* genes from all the *B. napus* accessions and 18 accessions maintained six *BnHR* genes (Supplementary Table S3). These data suggest that *BnHRs* derived from *BoHRs* are highly stable, whereas, *BnHRs* derived from *BrHRs* are less stable and may tolerate mutations.

### Genetic Variation at *BnHR*s

To dissect the genetic variation at *BnHR*s, we analyzed the nucleotide polymorphism of them using DnaSP version 5.10.1 (Rozas, 2009). Because no polymorphism was detected among amplicons of *BnHR*(*Bo*) from different accessions, we focused on *BnHR*(*Br*) genes for polymorphism analysis. As shown in **Table 1**, all alleles of the *BnHR*s have similar overall gene structure of two exons split by one intron, but are varied in length from 746 to 1148 bp. While the size of the two exons is quite similar, the single intron is quite different. The first exons are almost the same length of 296 bp, except of 293 bp for *BnHRd*(*Br*); whereas, the second exons are varied with the longest for *BnHRb*(*Br*) and *BnHRc*(*Br*) being 349 bp, the shortest for *BnHRd*(*Br*) being 319 bp, and the intermediate for *BnHRa*(*Br*) being 340 bp (**Table 1**). The intron is 107 bp for *BnHRa*(*Br*), 105 bp for *BnHRb*(*Br*) and *BnHRc*(*Br*), 533 bp for *BnHRd*(*Br*), respectively (**Table 1**). Genetic variation of different *BnHR*s is slightly different with the highest being *BnHRc*(*Br*), followed by *BnHRb*(*Br*); whereas, *BnHRa*(*Br*) and *BnHRd*(*Br*) are relatively conserved as indicated by Pi and Theta value (**Table 1**).

**Table 1:** Nucleotide polymorphism of Homologs of RPW8 from *B. napus*^∗^.

Locus (number of alleles)	Component	Location	Number of sites^a^	S	π	𝜃	Tajima’s D
BnHRa(Br) (8)	Total	1–748	746	5	0.00298	0.00247	0.88079
	Exon 1	1–296	296	1	0.00169	0.00124	0.98627
	Intron	297–405	107	1	0.00363	0.00344	0.15647
	Exon 2	406–748	340	3	0.00389	0.00322	0.79438
BnHRb(Br) (17)	Total	1–756	753	27	0.01055	0.01061	-0.02294
	Exon 1	1–296	296	8	0.00666	0.00799	-0.5915
	Intron	297–401	105	2	0.00658	0.00563	0.42027
	Exon 2	402–756	349	17	0.015	0.01429	0.19461
BnHRc(Br) (14)	Total	1–789	753	29	0.01144	0.01295	-0.49941
	Exon 1	1–296	296	14	0.01552	0.017	-0.35702
	Intron	297–434	105	3	0.00848	0.00898	-0.17279
	Exon 2	435–789	349	12	0.0089	0.01072	-0.67711
BnHRd(Br) (6)	Total	1–1161	1148	6	0.00238	0.00229	0.23001
	Exon 1	1–293	293	1	0.00114	0.00149	-0.93302
	Intron	294–839	533	4	0.00338	0.00329	0.14908
	Exon 2	840–1161	319	1	0.00186	0.00136	1.4451

*^∗^For Tajima’s D, no value is significant at 0.05 level (*P* > 0.10). S, number of variable sites; π, nucleotide diversity per site; 𝜃, Watterson’s nucleotide diversity estimator based on silent site. Tajima’s D is based on the differences between the number of segregating sites and the average number of nucleotide differences. ^a^Total number of sites exclude sites with gaps/missing data.*

To judge the type of natural selection of *BnHR*s, we calculated the rate of non-synonymous substitution (Ka) that causes an amino acid change and that of synonymous substitution (Ks) that does not, between *BnHR*(*Br*) and their *BrHR* ancestors at the coding region. As shown in Supplementary Table S4, the Ka/Ks ratios between most of the *BnHR* alleles and their ancestors were larger than 1, indicating positive selection on these alleles. Nevertheless, the Ka/Ks ratios between some *BnHR* alleles and their ancestors were assigned 99 in the program of PAML software, because these alleles did not have any synonymous substitution.

Polymorphism at the *BnHRa*(*Br*) locus is in the intermediate among the *BnHR* genes. We detected eight haplotypes at *BnHRa*(*Br*) locus from 44 *B. napus* accessions (**Table 1**; **Figure 1**). All alleles are different from their ancestor *BrHRa*. There were five nucleotide-segregating sites, two of which were singletons. Among the segregating sites, one occurred in the intron and four in the exons, three of which led to non-synonymous substitutions (**Figure 1**; **Table 1**). One SNP was detected in the first exon that did not change the amino acid residue, two in the intron with one T to G substitution in 27 accessions represented by N105-1 and Zh292, and one AT insertion in three accessions represented by R0 (**Figure 1**; **Supplementary Figure S1A**). There were three SNPs in the second exon that led to non-synonymous substitutions, one at nt position 495 with C to T point mutation in nine accessions resulting in the L130F substitution, one at nt 511 in all accessions with T to C point mutation resulting in the F135S substitution, and one at nt 575 with A to C mutation in 24 accessions resulting in the R156S substitution (**Figure 1**; **Supplementary Figure S1B**). Both F135S and R156S substitutions were converted to *BoHRa* at these sites (**Figure 1**; **Supplementary Figure S1B**). The distinguishable eight haplotypes encode four distinct BnHRa(Br) proteins (**Figure 1**; **Supplementary Figure S1**). Tajima’s D values are positive for the overall gene and each intron/exon structures (**Table 1**), implying balance selection. These data indicate that *BnHRa*(*Br*) is prone to evolving into *BnHRa*(*Bo*) and might have been subjected to balance selection.

Polymorphism at the *BnHRb*(*Br*) locus is high among the *BnHR* genes. In a previous report, two SNPs were reported between *BrHRb* and *BoHRb* (Xiao et al., 2004). In this study, we amplified *BnHRb*(*Br*) from 83 accessions representing 17 genotypes (**Figure 2**). Sequencing analysis revealed that there were 27 nucleotide-segregating sites with two sites in the intron and 25 sites in the exons, 19 of which are non-synonymous substitutions (**Figure 2**; **Table 1**). There are 19 SNPs resulting in aa substitutions, two and seven SNPs in intron and exon without aa changes (**Figure 2**). A deletion of AGT was detected in 25 accessions (**Figure 2**). Tajima’s D value is negative for the overall gene and the first exon, but positive for the intron and the second exon (**Table 1**), implying positive selection for this locus. Thus, the *BnHRb*(*Br*) locus is variable and might have been subjected to positive selection.

Polymorphism at the *BnHRc*(*Br*) locus is the highest among the *BnHR* genes with a Pi = 0.01144 (**Table 1**). In a previous report, eighteen SNPs were detected between *BrHRc* and *BoHRc* (Xiao et al., 2004). In this study, we detected 26 SNPs between *BnHRc*(*Br*) and its ancestor *BrHRc* from 74 accessions. In addition, an TA insertion in the intron was detected in 39 accessions and a GTC insertion in the second exon was detected in six accessions (**Figure 3**; **Supplementary Figure S2A**). Nineteen SNPs led to aa substitutions, of which eight converted to *BoHRc* (**Figure 3**; **Supplementary Figure S2B**), presumably caused by allele recombination between *BrHRc* and *BoHRc*. Moreover, a 29 bp insertion was found in nine accessions represented by N102-4 (**Figure 3**; **Supplementary Figure S2A**), indicating a common origin of these nine accessions. Tajima’s D values are negative for the overall gene and each exon/intron structures (**Table 1**), implying positive selection. Therefore, similar to *BnHRb*(*Br*), *BnHRc*(*Br*) is also quite variable and might have been subjected to positive selection.

Polymorphism at the *BnHRd* locus is low among the *BnHR* genes with the lowest Pi value, but comparable to that of the *BnHRa*(*Br*) locus (**Table 1**). Previously, *HRd* was found only in *B*. *rapa* (Xiao et al., 2004). In this study, we successfully amplified *BnHRd* from all tested accessions and discovered six types of variation from that of *BrHRd* (**Figure 4**). Most (78) accessions had three SNPs in the intron and one SNP at nt position 883 resulting in the I114L substitution. *BnHRd* from four accessions had three SNPs in the intron, and thus, had the same aa sequences as its ancestor *BrHRd*. The most varied *BnHRd* allele was detected in two accessions represented by DY-8AB that contained a SNP in the first exon leading to the A79T substitution, four SNPs in the intron of which three were the same mentioned above, one 10 bp and one TAA deletion in the intron (**Figure 4**). Tajima’s D value is positive for the overall gene, the intron and the second exon, but negative for the first exon (**Table 1**), implying positive selection for the first exon and balance selection for the intron and the second exon. Therefore, *BnHRd* is relatively conserved and might have been subjected to purifying selection.

### Recombination between Different *BnHR* Genes

**FIGURE 5:**
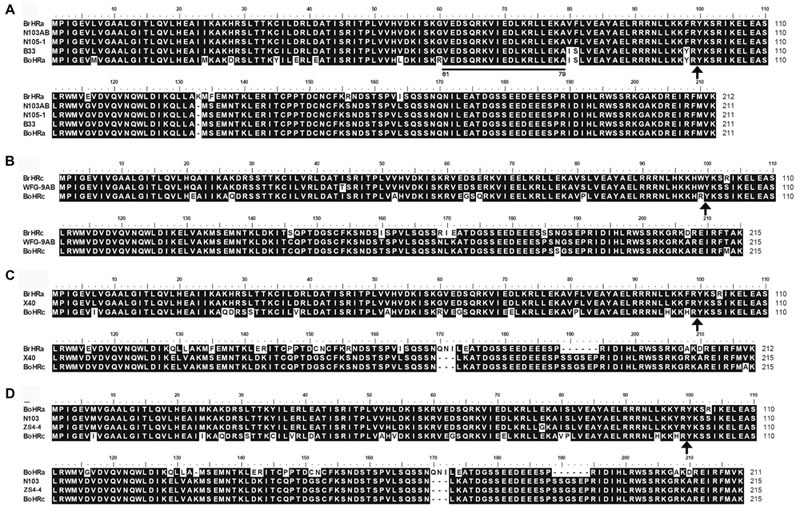
**Recombination between *BnHR* genes led to the formation of chimeric proteins.** The corresponding intron site was marked by an arrow. **(A)** Putative amino acid residues from the recombination between BrHRa and BoHRa forming two chimeric proteins with one in the accession B33 whose aa residue was identical to BrHRa from positions 1 to 79, while aa residues from positions 61 to 211 were identical to BoHRa. The underlined residues from 61 to 79 were identical to both BrHRa and BoHRa. The other recombination was detected in both accessions N103AB and N105-1 whose aa residues were identical to BrHRa in the first exon and identical to BoHRa in the second intron because the crossover site was in the intron (see **Supplementary Figure S3A**). **(B–D)** Putative amino acid residues from the recombination between BrHRc and BoHRc **(B)**, BrHRa and BoHRc **(C)**, and BoHRa and BoHRc **(D)** forming chimeric proteins with the first exon from one gene and the second exon from the other gene, respectively.

Intragenic recombination is one major mechanism for *R* gene evolution (Leister, 2004). During sequence analysis, we detected several recombination events between orthologs or between paralogs with the first half from one gene and the second half from the other gene. Specifically, we detected two recombination events between the two orthologs *BrHRa* and *BoHRa* that result in new alleles encoding two chimeric BnHRa proteins. One was presented in the accession B33 whose amino acid (aa) residues were identical to BrHRa from positions 1 to 79, while those from positions 61 to 211 were identical to BoHRa (**Figure 5A**). The underlined residues from 61 to 79 were identical to both BrHRa and BoHRa, indicating that this region may be the site of the crossover (**Supplementary Figure S3A**). The other recombination event was detected in both accessions N103AB and N105-1 whose aa residues were identical to BrHRa in the first exon and identical to BoHRa in the second exon, indicating that the crossover occurred in the intron (**Figure 5A**; **Supplementary Figure S3A**). The recombination detected in the accession WFG-9AB occurred between the two orthologs *BrHRc* and *BoHRc* leading to a new allele encoding a chimeric BnHRc protein combining the first exon of *BrHRc* and the second exon of *BoHRc* (**Figure 5B**; **Supplementary Figure S3D**). The other two types of recombination occurred between paralogs resulting in new *BnHR* genes with the first exon from one gene and the second exon from the other gene found in three accessions, including X40, N103, and ZS4-4 (**Figure 5**). The recombination detected in the accession X40 occurred between *BrHRa* and *BoHRc* leading to a new allele encoding a chimeric protein combining the first exon of *BrHRa* and the second exon of *BoHRc* (**Figure 5C**; **Supplementary Figure S3C**). The recombination detected in the accessions N103 and ZS4-4 occurred between *BoHRa* and *BoHRc* leading to a chimeric *BnHR* gene combining the first exon of *BoHRa* and the second exon of *BoHRc* (**Figure 5D**; **Supplementary Figure S3B**). Because the paralogs *HRa, HRb*, and *HRc* are tandemly arrayed in a syntenic chromosomal fragment in *B*. *rapa* and *B*. *oleracea*, it is very likely that *BnHR* genes have evolved through normal and uneven recombination between *BoHR* and *BrHR* orthologs as well as *HRa, HRb*, and *HRc* paralogs from both contributing genomes, resulting in new genes or gene loss.

### Ectopic Expression of *BnHR* Genes in *Arabidopsis* Led to Cell Death and Enhanced Resistance to Powdery Mildew

**FIGURE 6:**
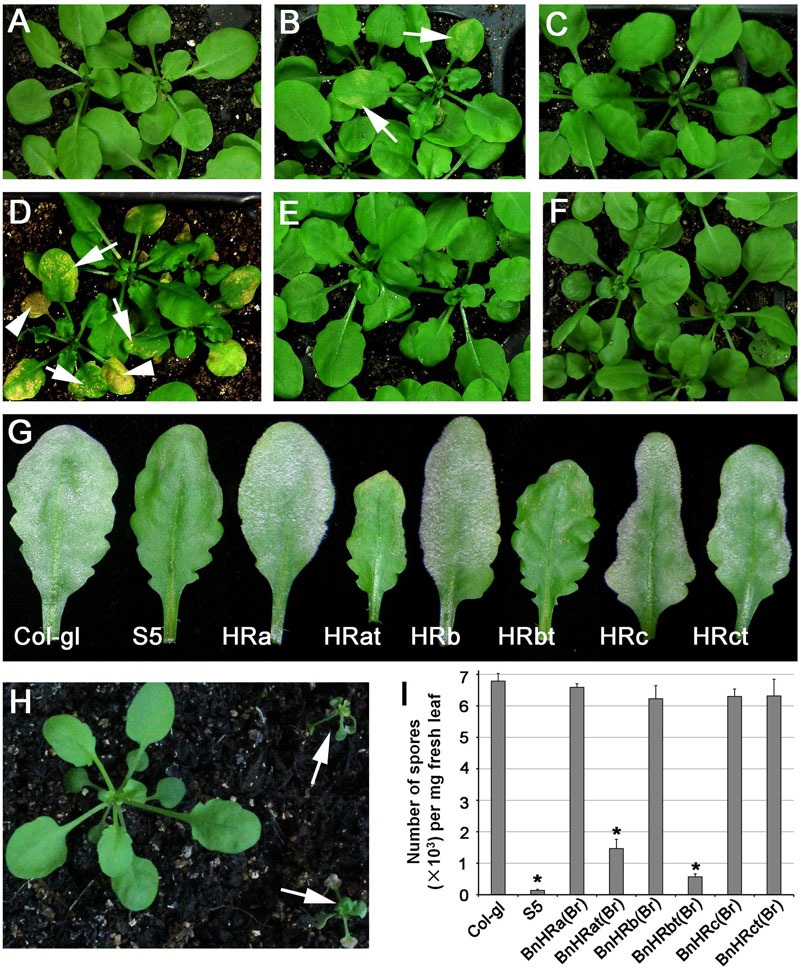
**Ectopic expression of *BnHR* genes leads to differential autoimmune activity and resistance to powdery mildew.** Representative plants expressing *BnHRa*(*Br*)-YFP **(A)**, *BnHRat*(*Br*)-YFP **(B)**, *BnHRb*(*Br*)-YFP **(C)**, *BnHRbt*(*Br*)-YFP **(D)**, *BnHRc*(*Br*)-YFP **(E)**, and *BnHRct*(*Br*)-YFP **(F)** from the *RPW8.2* promoter, respectively. Note that autoimmune activity was observed in some plants expressing the C-terminal truncated genes *BnHRat*(*Br*)-YFP or *BnHRbt*(*Br*)-YFP (arrows in **B,D**). Lesions were observed in young leaves (arrows in **D**) and expanded to whole leaves (arrowheads in **D**). **(G)** Representative leaves from the indicated lines to show powdery mildew disease phenotype. Col-gl and S5 were used as susceptible and resistant reference, respectively. Note that transgenic plants expressing the C-terminally truncated genes *BnHRat*(*Br*)-YFP and *BnHRbt*(*Br*)-YFP were resistant to powdery mildew. **(H)** Representative plants showed that transgenic plants expressing *BnHRd*-YFP were lethal at seedling stage (arrows). **(I)** Quantitative assay of disease susceptibility to *Gc* UCSC1 at 7 dpi. Values are means of three replications. Error bars indicate SD. Tukey’s Honestly Significant Difference test was carried out to determine the significance of differences between Col-gl and the indicated lines. Asterisks indicate significant difference at *P* < 0.0001.

The size of the RPW8 family members varies in length with HR3 being the longest and RPW8 the shortest (Xiao et al., 2004). This observation, together with results from our recent mutational analyses of RPW8.2 (Wang et al., 2013), suggests that acquiring a shorter C-terminus may contribute in part to the evolution of the resistance function of RPW8. Therefore, to evaluate the function of *BnHR* genes in disease resistance, we made two kinds of constructs with one expressing the full length protein and the other expressing the C-terminally truncated protein that was similar to RPW8 in length, and all were tagged with YFP at the C-terminus to aid the examination of protein expression and localization. The constructs were introduced into *Arabidopsis* accession Col-gl that is susceptible to powdery mildew and more than 15 independent T1 lines for each construct were examined for spontaneous cell death, which is indicative of auto-activated immunity, and resistance to powdery mildew by inoculating them with *G*. *cichoracearum* UCSC1. Our data showed that while transgenic lines expressing the full length proteins of BnHRa, BnHRb, and BnHRc did not show any obvious phenotypes, those expressing the C-terminally truncated version of both BnHRa (i.e., BnHRat-YFP) and BnHRb (i.e., BnHRbt-YFP) exhibited spontaneous cell death (**Figures 6A–F**). Next, we examined disease phenotype by inoculating *G. cichoracearum* UCSC1 on leaves of 5-weeks-old plants. We observed clear enhanced resistance in the transgenic plants expressing either BnHRat-YFP or BnHRbt-YFP (**Figure 6G**). Spore counting showed that the levels of fungal sporulation were significantly reduced in the transgenic lines expressing either BnHRat-YFP or BnHRbt-YFP, which were comparable to the resistant reference line S5 that contains *RPW8* (**Figure 6I**). Intriguingly, ectopic expression of BnHRd-YFP seemed to be lethal because the transgenic plants were dying at seedling stage (**Figure 6H**). These results suggest that BnHRat or BnHRbt may be functional to trigger cell death and to activate resistance against powdery mildew, while the full-length version of these two proteins are less potent or unable to function in *Arabidopsis*.

### Differential EHM-Targeting of BnHR-YFP Proteins

**FIGURE 7:**
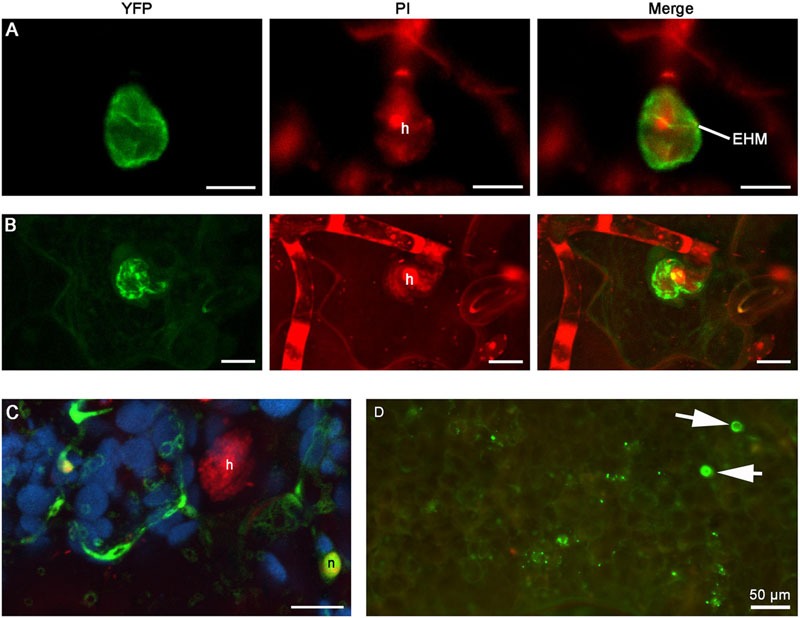
**Extra-haustorial membrane (EHM)-localization of BnHRa-YFP.** Confocal micrographs show the subcellular localization of the following BnHRa-YFP proteins. Yellow fluorescent protein (YFP)-tagged proteins were pseudo-colored green, whereas Propidium Iodide (PI) stained fungal structures were pseudo-colored red and auto-fluorescence from chloroplast was pseudo-colored blue. h, haustorium. EHM, extra-haustorial membrane. Size bars in **(A–C)**, 10 μm. **(A)** EHM-localization of BnHRa(Bo)-YFP. **(B)** BnHRa(Br)-YFP was mainly localized at the surface of a haustorium and in the cytoplasm of the cell. **(C)** BnHRat(Br)-YFP was localized in the cytoplasm and the nucleus (n) of the cell. **(D)** A representative epi-fluorescent micrograph shows that BnHRat(Br)-YFP was occasionally observed as haustorium-like objects (arrows).

**FIGURE 8:**
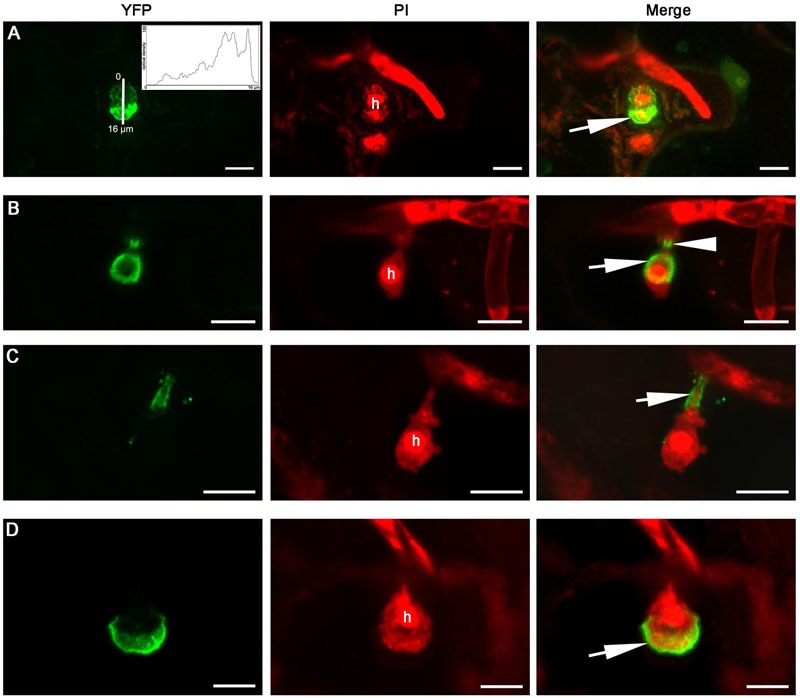
**Extra-haustorial membrane-localization of BnHRb-YFP.** Confocal micrographs show the subcellular localization of the following BnHRb-YFP proteins. Yellow fluorescent protein (YFP)-tagged proteins were pseudo-colored green and Propidium Iodide (PI) stained fungal structures were pseudo-colored red. h, haustorium. EHM, extra-haustorial membrane. Size bars, 10 μm. **(A)** BnHRb(Br)-YFP was localized at the surface of a haustorium and in the cytoplasm of the cell. Note the signal at the apical part of the haustorium (inset and arrow) is more intense. Inset shows the YFP signal intensity from 0 to 16 μm. **(B)** The C-terminally truncated version BnHRbt(Br)-YFP was localized at the EHM portion surrounding the basal half of the haustorium (arrow) and the haustorial neck (arrowhead). **(C)** BnHRbt(Br)-YFP was localized at the haustorial neck (arrow). **(D)** BnHRbt-m(Br)-YFP containing four aa substitutions, including S35C, V80A, V109A, and I115A in BnHRbt(Br), was localized at the apical part of the haustorium (arrow).

**FIGURE 9:**
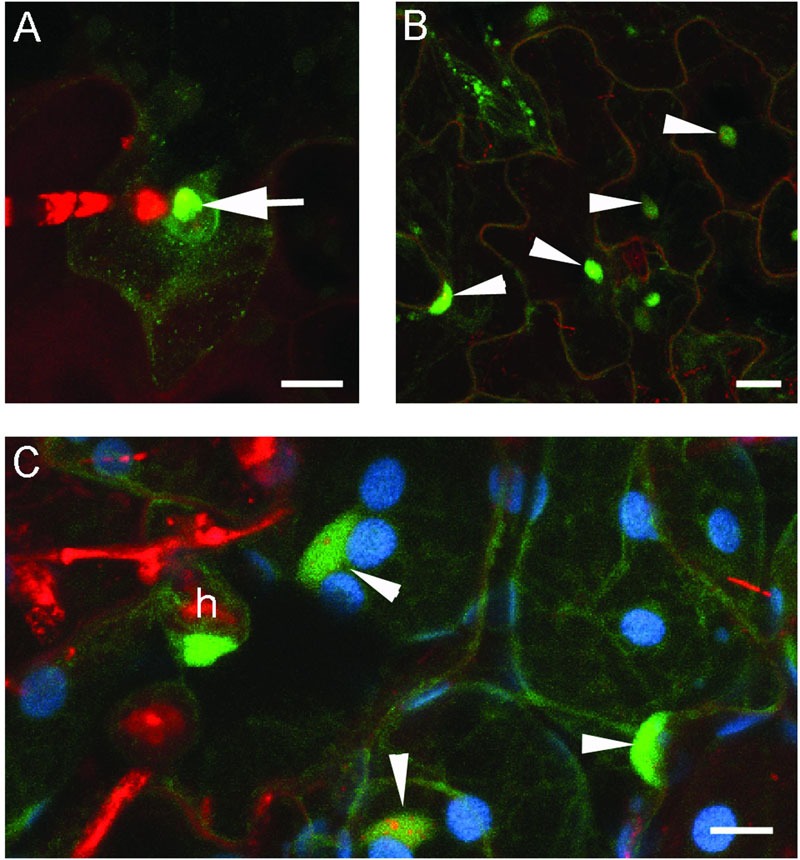
**Global localization of BnHRc-YFP.** Confocal micrographs show the subcellular localization of BnHRc-YFP proteins. Yellow fluorescent protein (YFP)-tagged proteins were pseudo-colored green, whereas, Propidium Iodide (PI) stained fungal structures were pseudo-colored red. h, haustorium. Size bars, 10 μm. **(A)** BnHRc(Br)-YFP localized in the cytoplasm but was enriched at the penetration site (Arrow). **(B)** BnHRc(Br)-YFP was localized in the nucleus (arrowheads) and the cytoplasm. **(C)** BnHRc(Bo)-YFP was localized in the nucleus (arrowheads) and the cytoplasm surrounding the haustorium (h) of powdery mildew.

When expressed in epidermal cells by the *RPW8.2* promoter, RPW8.1-YFP was also targeted to the EHM encasing the haustorium of powdery mildew (Wang et al., 2009; Ma et al., 2014). We asked whether BnHR proteins are localized to the EHM. To this end, we made transgenic *Arabidopsis* lines expressing each of the *BnHR* genes with YFP at the C-terminus from the *RPW8.2* promoter. The subcellular localization of each BnHR-YFP was examined at 2 days post-inoculation of *G. cichoracearum* SICAU1 (Zhang et al., 2015). Although, EHM-localization was detected for all the fusion proteins except for BnHRc-YFP (**Figures 7–9**), there were some differences in their localization patterns. While both BnHRa(Bo)-YFP and BnHRa(Br)-YFP were mainly distributed in the EHM encasing the haustorial complex, BnHRa(Br)-YFP was also found in the cytoplasm of the haustorium-invaded cell (**Figures 7A,B**). Similarly, the full length BnHRb(Br)-YFP was also localized to the EHM; but in some cases, we found higher YFP signal in the EHM portion surrounding the apical part of the haustorium distal to the haustorium neck, while weak YFP signal was also detectable in the cytoplasm of the cell (**Figure 8A**). Out of our expectation, the C-terminally truncated version of BnHRa, i.e., BnHRat(Br)-YFP was located in the cytoplasm (**Figure 7C**). However, we cannot exclude the possibility that BnHRat(Br)-YFP could target to EHM because we occasionally observed haustorial complex-like fluorescent objects (**Figure 7D**), although we failed to acquire any high-resolution images. Intriguingly, the C-terminally truncated version of BnHRb, i.e., BnHRbt(Br)-YFP, was found in the EHM portion surrounding the basal half of the haustorium or the haustorial neck (**Figures 8B,C**). More interestingly, when four site mutations were by chance introduced in the BnHRbt(Br)-YFP, including S35C, V80A, V109A and I115A, the mutant protein was located at the EHM portion surrounding the apical part of the haustorium (**Figure 8D**). However, we did not observe EHM-localization for both BnHRc(Br)-YFP and BnHRc(Bo)-YFP. Instead, BnHRc(Br)-YFP was globally located in the cell with enrichment at the penetration site (**Figure 9A**). In the uninvaded epidermal cells of the infected leaves, BnHRc(Br)-YFP was found in the cytoplasm and the nucleus (**Figure 9B**). Similar to BnHRc(Br)-YFP, BnHRc(Bo)-YFP was localized in the nucleus and the cytoplasm surrounding the haustorium (h) of powdery mildew (**Figure 9C**). However, we did not detect any signal for BnHRd-YFP. Collectively, these data indicate that homologs of RPW8 in *B. napus* may have functionally diverged in terms of protein localization with BnHRa and BnHRb being able to localize to the EHM and BnHRc to the penetration site in epidermal cells invaded by powdery mildew.

## Discussion

In this study, we determined the sequence polymorphisms of *RPW8* homologs (*BnHR*s) from the allotetraploid *B. napus* and assessed their role as resistance genes and protein localization in *Arabidopsis* through a transgenic approach. Our results indicate that multiple evolutionary mechanisms were involved in creating and maintaining *BnHR* genes in the *B. napus* genome and some *BnHR* genes may be valuable in engineering disease resistant plants because their ectopic expressions lead to cell death and resistance to powdery mildew. However, there is apparent sequence and likely functional divergence among these *BnHR* genes. In theory, there should be seven homologs of *RPW8* in *B. napus* given its allotetraploid nature from *B. rapa* and *B. oleracea*, because *B. rapa* contains four and *B. oleracea* contains three homologs of *RPW8* (Xiao et al., 2004). We were successful in identifying all these genes except *BoHRb* from 18 of the 88 *B. napus* accessions tested. Through heterologous expression in *Arabidopsis* from the *RPW8.2* promoter, we observed striking difference between different BnHR genes in their ability to activate cell death: while C-terminally truncated BnHRa and BnHRb, as well as full-length BnHRd could activate cell death in the absence of any pathogen, no obvious altered phenotypes were observed in *Arabidopsis* plants expressing full length BnHRa, BnHRb, BnHRc and the C-terminally truncated BnHRc (**Figure 6**). These observations imply that the full length BnHRa and BnHRb may be self-regulated with their C-termini inhibiting their activity. When the C-terminus is removed or perturbed, the protein is activated. This speculation is consistent with our earlier observation that some C-terminal six-aa (i.e., NAAIRS) replacement RPW8.2 mutants caused seedling-lethal cell death (Wang et al., 2013). Nevertheless, detailed analyses are required to understand how they are self-regulated.

Extra-haustorial membrane-localization and activation of haustorium-targeted defense is a unique feature of RPW8.2. We thus investigated if the BnHR proteins are also localized to the EHM. While our localization analysis suggest that BnHRa-YFP and BnHRb-YFP appear to be localized to the EHM, the EHM-targeting efficiency or specificity might be lower than that of RPW8 because the haustoria labeled by YFP were hard to be observed, and YFP signal could be observed in papillae or the cytoplasm (**Figures 7–9**). These observations imply that either these BnHR proteins may have evolved divergent localization properties or these proteins are not completely compatible with the trafficking machinery for precise EHM-localization in *Arabidopsis* epidermal cells. Thus, functional validation in *B. napus*, e.g., gene knockout by CRISPR/cas9 technology (Basak and Nithin, 2015), is required to determine the authentic roles of these *BnHR* genes in disease resistance.

What fate *R* genes may face after diploidization of a hybrid is an interesting question. In this study, although we did not physically defined the chromosomal location of all the seven possible *BnHR* genes, our sequence results based on PCR amplification should still offer some insight into this question. Our data showed that while *BnHRa*(*Bo*) is highly conserved in *B. napus* as indicated by 100% sequence identity of all amplicons from 77 accessions to *BoHRa, BnHRa*(*Br*) is more variable, because all amplified *BnHRa*(*Br*) alleles are different from *BrHRa* (**Figure 1**). Nevertheless, the variation seems to be restricted, because among 44 amplicons, we detected five sites variation from *BrHRa*, only three of which resulted in amino acid substitutions and two of the substitutions were converted to BoHRa (**Figure 1**). Interestingly, despite repeated efforts, we failed to amplify either *BnHRa*(*Bo*) or *BnHRa*(*Br*) in some *B. napus* accessions (Supplementary Table S3), suggesting that these two orthologs might have been lost in *B. napus* genome after diploidization. However, the mechanism for gene-loss remains an open question.

Intriguingly, all amplified *BnHRb* alleles appeared to be derived from *BrHRb*, because they are identical to *BrHRb* at the two nucleotide sites that differ from *BoHRb* (**Figure 2**). There are some possibilities to explain why we did not identify *BoHRb* in *B. napus*. First, *BoHRb* might be lost in *B. napus*. Second, *BrHRb* is dominant over *BoHRb* in PCR amplification. Third, the two sites distinguishing *BrHRb* and *BoHRb* were all converted to *BrHRb*. Regardless the origin, *BnHRb* seems to be highly variable as indicated by the existence of abundant allelic polymorphic sites and non-synonymous substitutions (**Table 1**; **Figure 2**). It is possible that strong selection has been acting on this gene locus for novel function in defense.

In contrast to the seemingly selective maintenance of *BnHRb*(*Br*), both *BnHRc*(*Br*) and *BnHRc*(*Bo*) could be maintained in *B. napus*. More than half (46) of the tested *B. napus* accessions contain *BnHRc* from both *B. rapa* and *B. oleracea*, despite that many accessions seem to contain one copy of *BnHRc* from either *B. rapa* or *B. oleracea* (Supplementary Table S3). Interestingly, while the copy from *B. oleracea* is highly conserved and its nucleotide sequences are identical to its ancestor *BoHRc*, the copy from *B. rapa* seems to have been under positive selection as indicated by the negative value of Tajima’s D and a high rate of non-synonymous substitutions between the amplified *BnHRc*(*Br*) alleles and *BrHRc* (**Table 1**; **Figure 4**; Supplementary Table S4). This observation nicely conforms to the pattern of neofunctionalization after gene duplication, with one copy retaining the original function and the other being diverged for novel function.

Consistent with the previous report that *BrHRd* is a singleton at a separate locus in *B*. *rapa* and is also most similar to *HR3* (Xiao et al., 2004), *BnHRd*(*Br*) has been maintained in all tested *B. napus* accessions. This gene seems less variable compared with the other *BnHR* genes: there are only six segregating sites among the 88 *B. napus* accessions, of which two sites result in amino acid substitution (**Table 1**; **Figure 4**), suggesting that *BnHRd*(*Br*) is relatively more conserved in function. This speculation is further confirmed by the phenotypes of transgenic *Arabidopsis* plants expressing BnHRd-YFP being seedling-lethal (**Figure 6H**), similar to those expressing HR3-YFP (Berkey et al., unpublished data).

Recombination accounts for a major force for resistance gene evolution (Leister, 2004). Not surprisingly, we detected a few events of intragenic recombination between different pairs of orthologs and paralogs in the *BnHR* gene loci, including three events between *BrHRa* and *BoHRa*, one between *BrHRc* and *BoHRc*, one between *BrHRa* and *BoHRc*, one between *BoHRa* and *BoHRc* (**Figure 5**). The latter two non-allelic recombination could in particular explain the loss of *BoHRb* genes in the *B. napus* genome: *HRb* locates between *HRa* and *HRc*, when crossover occurs between *HRa* and *HRc*, the interval region containing *HRb* could be lost after recombination.

Taken together, our data suggest that in the *B. napus* genome, the *BrHR* copy of *HRa, HRb* and *HRc* from *B. rapa* tends to incorporate sequence variation, while the copy from *B. oleracea* is highly conserved, despite that either copy could undergo gene loss in *B. napus* after diploidization from the hybrid of *B. rapa* and *B. oleracea*. In addition, our analysis of the allelic polymorphism at the *BnHR* genes suggest that multiple evolutionary events, including gene loss, point mutation, insertion, deletion and intragenic recombination contribute to sequence and possible function diversification of the *BnHR* copy from *B*. *rapa*.

## Author Contributions

QL, JL, J-LS, X-FM, RB, and T-TW conducted the experiments. HY and Y-ZN provided *B. napus* seeds and conducted the field experiment. JF, YL, and SX supervised the study and edited the manuscript. W-MW coordinated the overall study and wrote the manuscript.

## Conflict of Interest Statement

The authors declare that the research was conducted in the absence of any commercial or financial relationships that could be construed as a potential conflict of interest.

## Supplementary Material

FIGURE S1 **Alignment of *BnHRa*(*Br*) against *BoHRa/BrHRa*. (A)** Nucleotide sequences of *BnHRa*(*Br*) aligned with *BoHRa* and *BrHRa*. Polymorphism sites from *BnHRa*(*Br*) amplified from 44 accessions were marked with ^∗^ and those lends to amino acid converted to those in BoHRa were marked with #. The intron borders were marked with ▾. Polymorphism sites between *BoHRa* and *BrHRa* were in black-white letters. **(B)** Alignment of amino acid residues. Three amino acid substitutions were detected in BnHRa(Br) from 44 accessions. One was L to F alteration at the aa position 130 (arrow) and two were altered to aa residue as in BoHRa at positions 135 and 156 (arrowheads), respectively.

FIGURE S2 **Alignment of *BnHRc*(*Br*) against *BrHRc*. (A)** Nucleotide sequences of *BnHRc*(*Br*) aligned with *BrHRc* and *BoHRc*. Polymorphism sites from *BnHRc*(*Br*) amplified from 63 accessions were marked with ^∗^ and those lends to amino acid substitutions were marked with arrows. The intron borders were marked with ▾. Polymorphism sites between *BoHRc* and *BrHRc* were in black-white letters. The underlined nucleotides including three codons (i.e., AGT AGT CTC) in the allele from six accessions represented by MY15-1-1 because of the insertion of GTC, but not TAG. **(B)** Alignment of amino acid residues. Six amino acid substitutions in BnHRc(Br) were converted into BoHRc (#).

FIGURE S3 **DNA sequence alignment to show the recombination between different *BnHR* genes.** Intron borders were marked with ▾ and possible crossover regions were underlined. **(A)** Recombination between *BrHRa* and *BoHRa* in three *B. napus* accessions. One crossover region possibly located in the first exon between positions 181 and 237 from accession B33. The other crossover region might be in the intron between 317 and 341 or 389, because there were one SNP at 342 and one nucleotide deletion at 347 (arrows) that could be due to sequence diversification after recombination. **(B)** Recombination between *BoHRa* and *BoHRc* in two accessions. Crossover site was possibly located in the intron from 310 to 315. Four SNPs were also detected (arrows). **(C)** Recombination between *BrHRa* and *BoHRc* in one accession. Crossover site was possibly occurred in the intron from positions 300 to 314. **(D)** Recombination between *BrHRc* and *BoHRc* in one accession. Crossover site was possibly occurred in the intron from positions 296 to 334. There were two SNPs equal to *BrHRc* in the second exon (arrows).
