# Supplementary figures and images for: Multiple Evolutionary Events Involved in Maintaining Homologs of Resistance to Powdery Mildew 8 in Brassica napus
Source: Front Plant Sci. 2016 Jul 21;7:1065. doi: 10.3389/fpls.2016.01065 (PMC4955382; doi:10.3389/fpls.2016.01065)

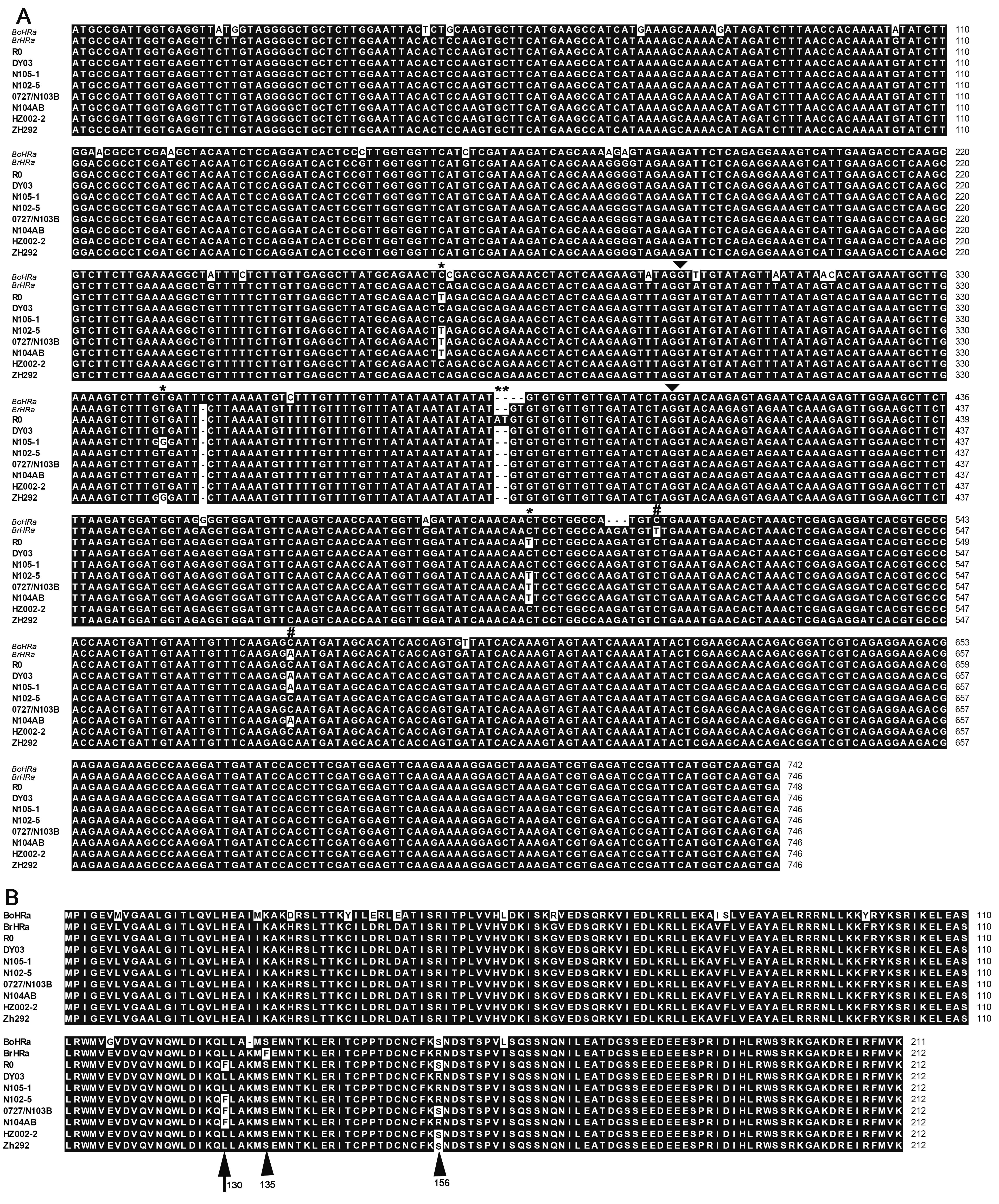

Supplement: FIGURE S1 — Alignment of BnHRa(Br) against BoHRa/BrHRa. (A) Nucleotide sequences of BnHRa(Br) aligned with BoHRa and BrHRa. Polymorphism sites from BnHRa(Br) amplified from 44 accessions were marked with ∗ and those lends to amino acid converted to those in BoHRa were marked with #. The intron borders were marked with ▾. Polymorphism sites between BoHRa and BrHRa were in black-white letters. (B) Alignment of amino acid residues. Three amino acid substitutions were detected in BnHRa(Br) from 44 accessions. One was L to F alteration at the aa position 130 (arrow) and two were altered to aa residue as in BoHRa at positions 135 and 156 (arrowheads), respectively. [file image_1.JPEG]

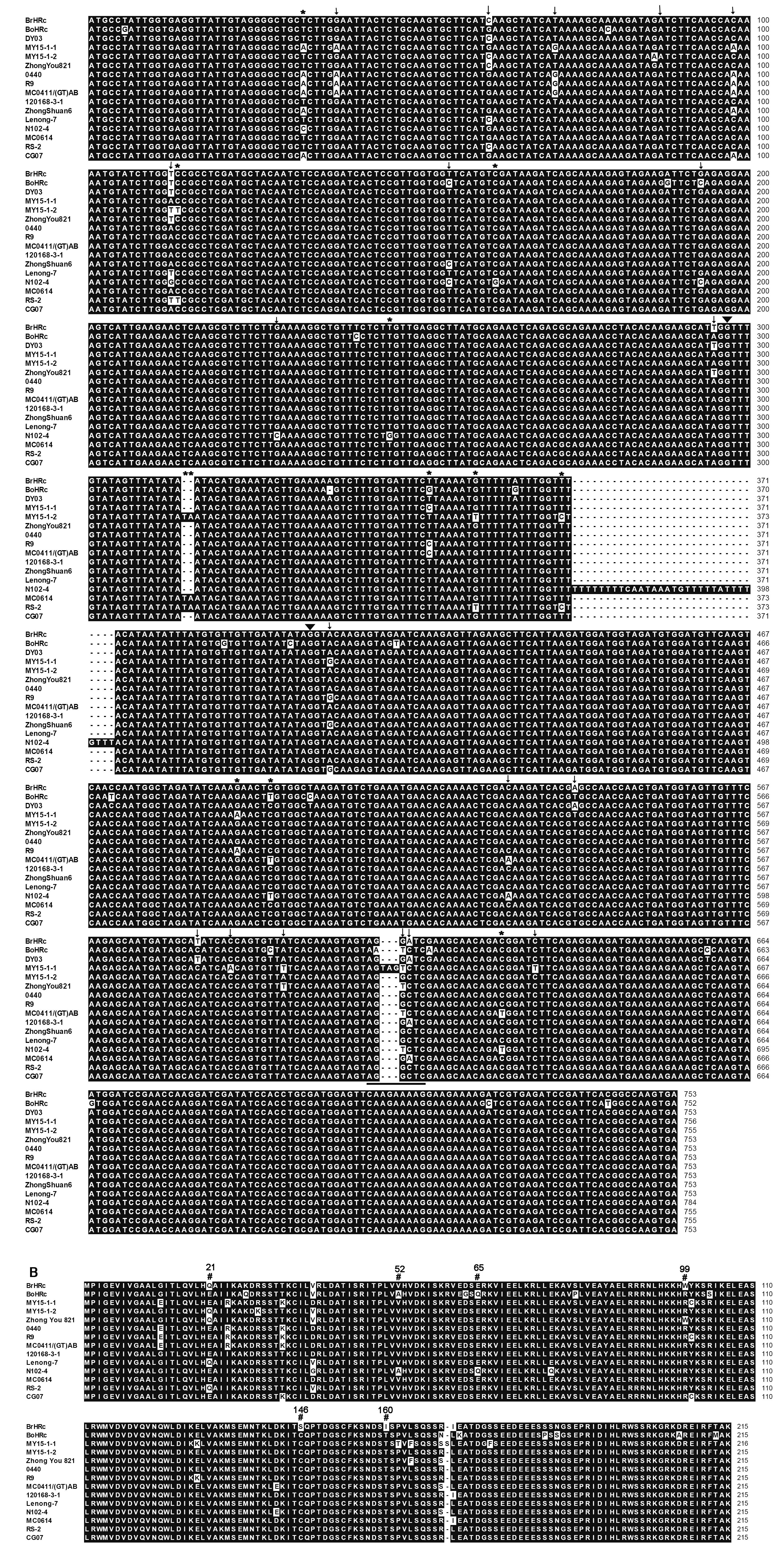

Supplement: FIGURE S2 — Alignment of BnHRc(Br) against BrHRc. (A) Nucleotide sequences of BnHRc(Br) aligned with BrHRc and BoHRc. Polymorphism sites from BnHRc(Br) amplified from 63 accessions were marked with ∗ and those lends to amino acid substitutions were marked with arrows. The intron borders were marked with ▾. Polymorphism sites between BoHRc and BrHRc were in black-white letters. The underlined nucleotides including three codons (i.e., AGT AGT CTC) in the allele from six accessions represented by MY15-1-1 because of the insertion of GTC, but not TAG. (B) Alignment of amino acid residues. Six amino acid substitutions in BnHRc(Br) were converted into BoHRc (#). [file image_2.JPEG]

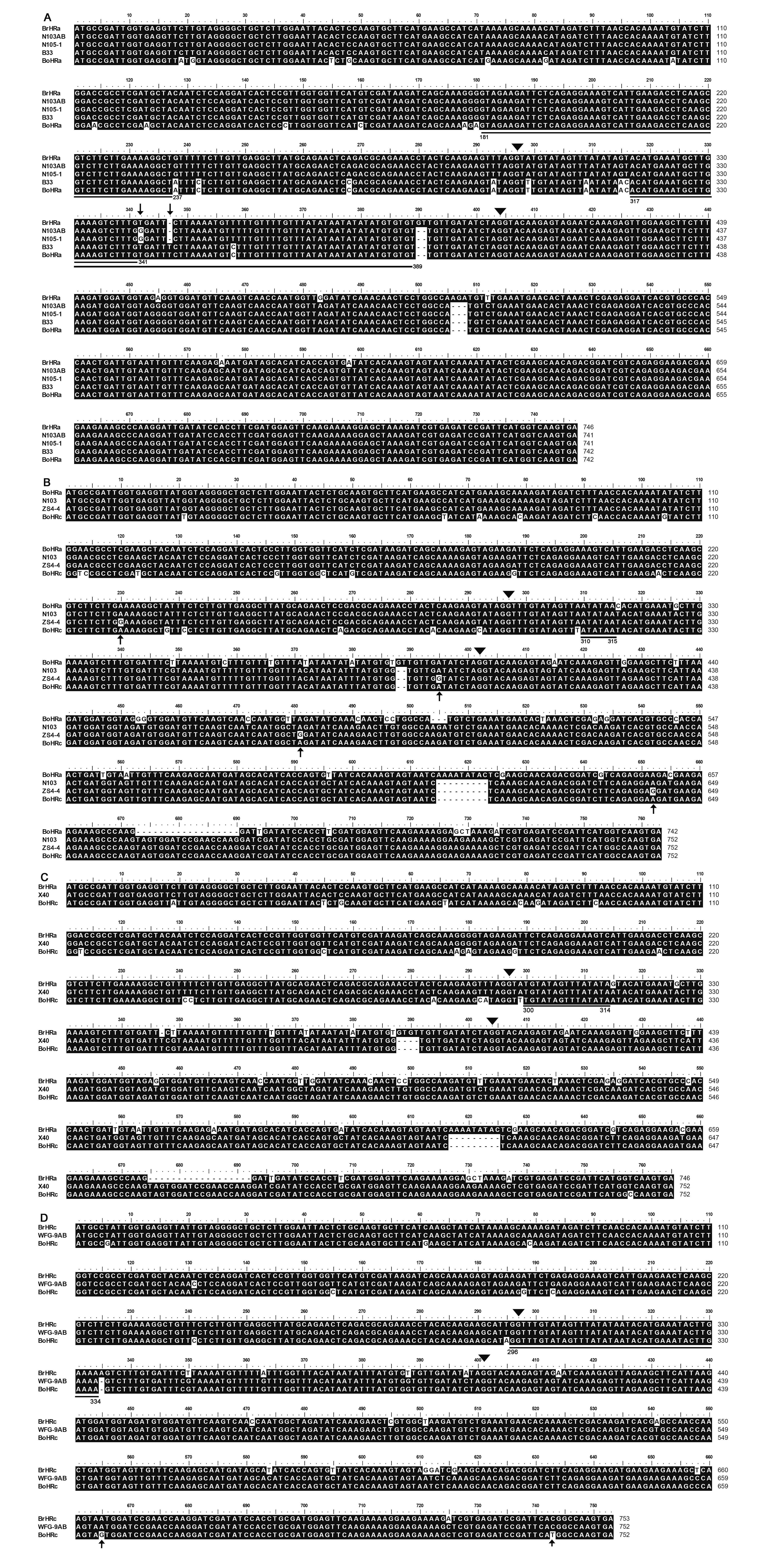

Supplement: FIGURE S3 — DNA sequence alignment to show the recombination between different BnHR genes. Intron borders were marked with ▾ and possible crossover regions were underlined. (A) Recombination between BrHRa and BoHRa in three B. napus accessions. One crossover region possibly located in the first exon between positions 181 and 237 from accession B33. The other crossover region might be in the intron between 317 and 341 or 389, because there were one SNP at 342 and one nucleotide deletion at 347 (arrows) that could be due to sequence diversification after recombination. (B) Recombination between BoHRa and BoHRc in two accessions. Crossover site was possibly located in the intron from 310 to 315. Four SNPs were also detected (arrows). (C) Recombination between BrHRa and BoHRc in one accession. Crossover site was possibly occurred in the intron from positions 300 to 314. (D) Recombination between BrHRc and BoHRc in one accession. Crossover site was possibly occurred in the intron from positions 296 to 334. There were two SNPs equal to BrHRc in the second exon (arrows). [file image_3.JPEG]
